# Longitudinal Relationship Between Self-Esteem and Academic Self-Efficacy Among College Students in China: Evidence From a Cross-Lagged Model

**DOI:** 10.3389/fpsyg.2022.877343

**Published:** 2022-05-23

**Authors:** Yunfeng Luo, Wenjuan Gao, Xinqiao Liu

**Affiliations:** ^1^School of Public Management, University of Electronic Science and Technology of China, Chengdu, China; ^2^Institute of Higher Education, Beihang University, Beijing, China; ^3^School of Public Administration, Beihang University, Beijing, China; ^4^School of Education, Tianjin University, Tianjin, China

**Keywords:** academic self-efficacy, self-esteem, gender differences, cross-lagged models, mental health

## Abstract

The present study aimed to investigate the associations between self-esteem and academic self-efficacy among Chinese college students. Descriptive statistics showed that on average, students’ academic self-efficacy experienced a downward trend in the first 3 years before rising slightly in the graduation year, and that male students had higher academic self-efficacy than females in the first 2 years, whereas female students’ academic self-efficacy surpassed their male counterparts in the latter years. There were significant, positive associations between the two variables. With cross-lagged analysis, we found that students’ self-esteem significantly predicted their subsequent academic self-efficacy from the freshman to the junior years, and the effects among male students endured longer and stronger. Implications of the findings were discussed.

## Introduction

Academic success has been regarded as the top priority for school education. Educational challenges or pressures may pose grave threats to students’ development, or even lead to school dropouts, and have long-term effects on their future careers (Entwisle et al., 2001; Ansary and Luthar, 2009). There is increasing recognition that academic self-efficacy plays a crucial role in students’ academic achievement (Pajares and Schunk, 2001; Huang, 2012; Jiang et al., 2014; Suprayogi et al., 2019). Academic self-efficacy is widely recognized as an individual’s views on their abilities to produce a specific level of academic achievement (Bandura et al., 1999). Previous studies found that the academic self-efficacy of students positively affected their engagement in studies (Ahmed et al., 2018), which serves as a determining factor for academic achievement (Kuh et al., 2006). Meanwhile, some researchers asserted that students with high academic self-efficacy showed stronger resilience in front of adversity, and persevered longer confronted with challenges (Klassen and Usher, 2010). In this way, they undoubtedly attain high academic performance with relentless efforts. Furthermore, some evidence showed the reciprocity between academic self-efficacy and educational achievement that the academic self-efficacy of students exerted positive impacts on their educational achievement, which, in return, further enhance their academic self-efficacy (Shea and Howell, 2000; Talsma et al., 2018).

A strand of the literature focused on the relationship between self-esteem and academic self-efficacy among students in high school and universities, and has reached the consensus that there was a significant positive correlation between students’ self-esteem and academic self-efficacy in different contexts such as the United States, the United Kingdom, China, and the United Arab Emirates (Lane et al., 2004; Afari et al., 2012; Di Giunta et al., 2013; Mao et al., 2020).

Furthermore, self-esteem has been confirmed to have a one-way impact on academic self-efficacy through promoting individuals’ initiatives (Lin et al., 2014). Self-esteem, as a perception of oneself, can bring in effects to the real world even the perception may not be correct (Robert and Lenore, 1968). Thus, the self-esteem of students is of great significance to their success, even their self-recognition may be exaggerated or undervalued. Self-esteem was considered to have relatively stable effects on fostering initiative and enhancing the sensation of pleasure for individuals (Baumeister et al., 2003). For instance, the extant empirical research has provided strong evidence that students’ self-esteem and academic achievement are positively related (Saadat et al., 2012; Arshad et al., 2015; Cvencek et al., 2018; Li et al., 2018). Moreover, self-esteem was found to exert an impact on people’s life satisfaction (Joshanloo and Afshari, 2011; Kong et al., 2012; Hawi and Samaha, 2017).

In general, although the associations between self-esteem and academic self-efficacy have been extensively explored, few studies examined the directionality of their relationship (Ahmadi, 2020). The present study aimed to fill the research gap by investigating the prospective associations between self-esteem and academic self-efficacy among college students in China over 4 years. First, understanding the directionality of the relationship between self-esteem and academic self-efficacy can be informative for colleges and universities to make more targeted interventions in student development. Second, given that Chinese undergraduate students often suffer from adaptation problems after entering universities (Kirkpatrick and Zang, 2011), proper interventions play a vital role to make them acclimatize to college life more quickly. The extant literature has presented strong evidence on the one-way effect of self-esteem on academic self-efficacy under various contexts (Lin et al., 2014), and in accordance with the proactive motivation model by Parker et al. (2010), self-esteem may be among the determining factors for academic self-efficacy. Thus, we proposed the first hypothesis of this study.

Hypothesis I: The self-esteem of Chinese college students can positively predict their subsequent academic self-efficacy over the four academic years.

By contrast, there has been little evidence concerning the effects of academic self-efficacy on self-esteem. The study assumed that academic achievement, rather than academic self-efficacy, may be more closely related to the levels of self-esteem among students. The outstanding academic performance of students may predict high self-esteem (Harris, 2009). In this regard, we presented the second hypothesis.

Hypothesis II: Students’ academic self-efficacy had no lagged impact on their self-esteem.

## Methods

### Participants

Participants in this study were 2473 full-time students (1166 females and 1307 males) admitted into 15 public universities in 2008 at Beijing in China, belonging to different fields of specialization such as science and engineering, social sciences, and humanities. More detailed information on the data was elaborated in Gao et al. (2020, 2022) and Liu et al. (2019a,b). The Beijing College Students Panel Survey (BCSPS) followed these students over the four academic years and collected their information including their self-esteem and academic self-efficacy yearly. The dropouts in the sophomore, junior, and senior years accounted for 4.73, 5.34, and 9.42%, respectively, and there were no significant differences in a series of variables between the dropouts and the completers. For instance, the completers were not significantly different in academic self-efficacy levels from the dropouts of the second (*t* = −0.219, *p* = 0.827), the third (*t* = −0.656, *p* = 0.512), and the fourth year (*t* = −1.060, *p* = 0.289).

### Power Consideration

The effect sizes of the present study were expected to fall into the interval between 0.2 and 0.5 based on relevant studies (Lane et al., 2004; Afari et al., 2012; Di Giunta et al., 2013; Mao et al., 2020). A *post hoc* power analysis with GPower 3.1.9.7 showed that our sample size of 2473 provided a sufficient power of 100% to estimate either an effect of 0.2 or an effect of 0.5 at α = 0.05. Furthermore, a sensitivity power analysis for the cross-lagged model indicated that our sample could detect effects higher than 0.05 with a one-tailed alpha of 5% and the power at 0.8.

### Measures

#### Academic Self-Efficacy

The academic self-efficacy of college students in this study was assessed with the 5 items of the Pattern of Adaptive Learning Scales’ (PALS) Academic Efficacy subscale, which estimated students’ perceptions of their academic competence (Midgley et al., 2000). Participants reported the extent to which they believed each item was true of them on a 1 (Not at all true) to 5 (Very true) scale, and their responses were summed to yield the global academic self-efficacy score ranging from 5 to 25. The reliability coefficient (α) of academic efficacy from the freshman to the senior year was 0.866, 0.853, 0.896, and 0.901, respectively, revealing good measurement reliability.

#### Self-Esteem

The 10-item Rosenberg self-esteem scale was widely adopted to measure general self-esteem (Rosenberg, 1965), which has been evidenced for the invariance of construct in the Chinese context, and this study used a 5-point Likert scale ranging from 1 (Strongly Disagree) to 5 (Strongly Agree). The internal consistency was satisfactory, with coefficients of scale reliability α varying from 0.878 to 0.887 during the four academic years.

## Results

### Descriptive Statistics and Correlation Analysis of Self-Esteem and Academic Self-Efficacy

On average, the score of academic self-efficacy among Chinese college students experienced a downward trend from 18.136 (SD = 4.372) in the freshman year to 17.267 (SD = 4.552) in the junior year, before rising slightly to 17.488 (SD = 4.157) in the senior year. Similarly, the mean scores of female and male students gradually declined from 17.72 (SD = 4.458) and 18.507 (SD = 4.261) to 17.445 (SD = 4.362) and 17.11 (SD = 4.711), respectively, in the first 3 years, after which they grew to 17.673 (SD = 3.956) and 17.322 (SD = 4.325) separately. Furthermore, male students scored higher in academic self-efficacy than females in the first 2 years, whereas the academic self-efficacy of female students surpassed their male counterparts in the latter 2 years, indicating that there existed gender differences in academic development. Additionally, the description of self-esteem for the sample may refer to previous research (Gao et al., 2022), which indicated that the average levels of self-esteem among both female and male students gradually declined over time, and female students had higher self-esteem than males.

The correlation analysis among the measures showed that the academic self-efficacy among college students, either females or males, was significantly positively associated with their levels of self-esteem both in the same academic year and across years (*p* < 0.01) (see Table 1). In addition, the academic self-efficacy in different years correlated positively (*p* < 0.01), and self-esteem of the 4 years also correlated strongly (*p* < 0.01).

**TABLE 1:** Correlation coefficients between self-esteem and academic efficacy.

Group	Year	Variables	1	2	3	4	5	6	7	8
All	Year 1	1. Self-esteem	1							
		2. Academic efficacy	0.407*	1						
	Year 2	3. Self-esteem	0.597*	0.289*	1					
		4. Academic efficacy	0.351*	0.551*	0.434*	1				
	Year 3	5. Self-esteem	0.433*	0.209*	0.518*	0.270*	1			
		6. Academic efficacy	0.264*	0.374*	0.292*	0.454*	0.592*	1		
	Year 4	7. Self-esteem	0.349*	0.203*	0.433*	0.253*	0.486*	0.304*	1	
		8. Academic efficacy	0.206*	0.324*	0.250*	0.408*	0.271*	0.445*	0.566*	1
Female	Year 1	1. Self-esteem	1							
		2. Academic efficacy	0.425*	1						
	Year 2	3. Self-esteem	0.626*	0.312*	1					
		4. Academic efficacy	0.383*	0.565*	0.460*	1				
	Year 3	5. Self-esteem	0.451*	0.232*	0.513*	0.319*	1			
		6. Academic efficacy	0.302*	0.408*	0.320*	0.503*	0.638*	1		
	Year 4	7. Self-esteem	0.395*	0.230*	0.506*	0.329*	0.530*	0.363*	1	
		8. Academic efficacy	0.243*	0.340*	0.290*	0.465*	0.340*	0.510*	0.565*	1
Male	Year 1	1. Self-esteem	1							
		2. Academic efficacy	0.399*	1						
	Year 2	3. Self-esteem	0.574*	0.278*	1					
		4. Academic efficacy	0.325*	0.533*	0.417*	1				
	Year 3	5. Self-esteem	0.417*	0.200*	0.520*	0.235*	1			
		6. Academic efficacy	0.233*	0.356*	0.268*	0.420*	0.555*	1		
	Year 4	7. Self-esteem	0.311*	0.198*	0.375*	0.201*	0.446*	0.254*	1	
		8. Academic efficacy	0.174*	0.321*	0.217*	0.368*	0.214*	0.394*	0.564*	1

*The asterisk symbol stands for 5% significance level.*

### Cross-Lagged Panel Analysis Between Self-Esteem and Academic Self-Efficacy

**FIGURE 1:**
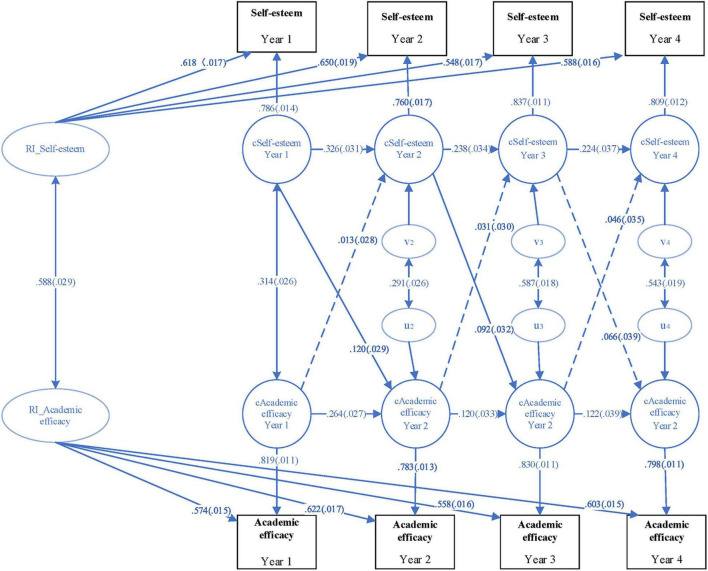
A four-wave cross-lagged panel model with random intercepts (all samples).

**FIGURE 2:**
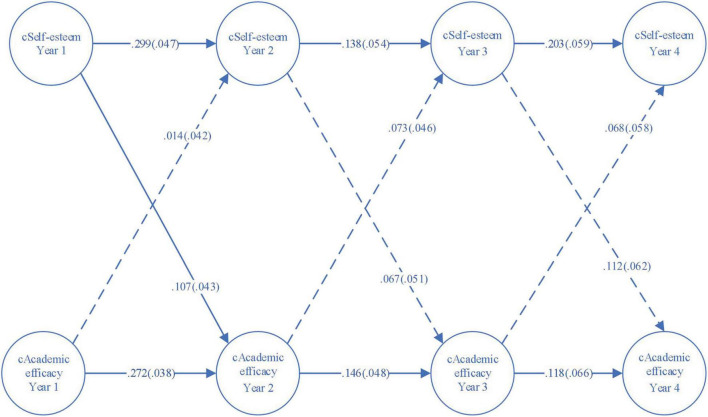
A four-wave cross-lagged panel model with random intercepts (female sub-samples).

**FIGURE 3:**
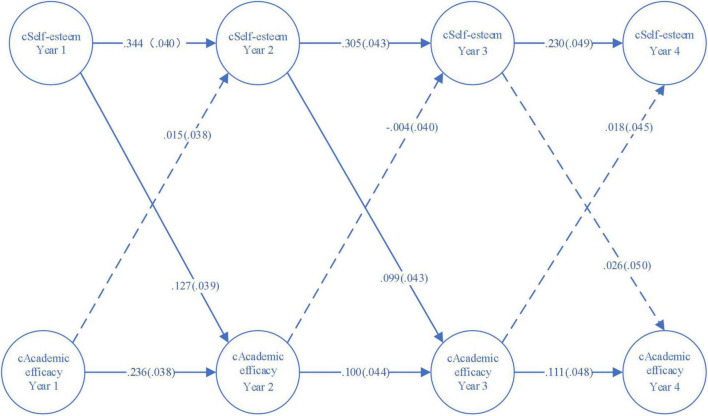
A four-wave cross-lagged panel model with random intercepts (male sub-samples).

A four-wave random intercepts cross-lagged model was employed to describe the prospective effects between self-esteem and academic self-efficacy across years among the whole population of students [root mean square error of approximation (RMSEA) = 0.034, 90% CI = (0.022, 0.046); CFI = 0.996, TLI = 0.989; standardized root mean square residual (SRMR) = 0.019] as demonstrated in Figure 1. Figure 2 presents the model for female sub-sample [RMSEA = 0.046, 90% CI = (0.029, 0.064); CFI = 0.994, TLI = 0.981; SRMR = 0.03] and Figure 3 the model for male sub-sample [RMSEA = 0.024, 90% CI = (0.0, 0.043); CFI = 0.998, TLI = 0.994; SRMR = 0.016]. Each fit statistics of the models to the data met the conventional criteria for a good fitting model. In the three figures, the RI-self-esteem stands for random intercept for self-esteem, and the RI_academic self-efficacy represents random intercept for academic self-efficacy. Besides, the solid lines in the three figures show statistically significant relationships while the dashed lines indicate that the relationships are not statistically significant.

As shown in Figure 1, students’ self-esteem and academic self-efficacy levels both showed strong stability, and their autoregressive standardized path coefficients varied from 0.120 to 0.264 (*p* < 0.01) and from 0.224 to 0.326 (*p* < 0.01), respectively. After controlling the autoregressive effects, students’ self-esteem in the first year had a significantly positive effect on their academic self-efficacy in the second year (standardized coefficient = 0.120, *p* < 0.01); likewise, the self-esteem of sophomore students also significantly predicted their academic self-efficacy in the junior year (standardized coefficient = 0.092, *p* < 0.01). This lagged positive impact also existed from the junior to the senior years, though not statistically significant (standardized coefficient = 0.066, *p* = 0.085). The results indicated that the prospective effects of self-esteem on academic self-efficacy among college students persisted over years though weakening. Conversely, there were no significant impacts from students’ academic self-efficacy to their self-esteem.

Figures 2, 3 demonstrated the reciprocal relations between self-esteem and academic self-efficacy for female and male students. The stability effects of both variables were all highly significant (*p* < 0.05), with the exception of the stability effects among females leading from the juniors’ academic self-efficacy to the seniors’ academic self-efficacy (*p* = 0.072). With the autoregressive effects being controlled, the self-esteem of both female and male students exerted subsequent impacts on their academic self-efficacy, yet the impacts among females were only statistically significant from the freshman year to the sophomore year (standardized coefficient = 0.107, *p* < 0.05), while the prospective effects among males were significant from the first year to the second year (standardized coefficient = 0.127, *p* < 0.01), and from the second year to the third year (standardized coefficient = 0.099, *p* < 0.05).

In summary, the self-esteem and academic self-efficacy of Chinese college students presented strong stability over the four academic years. Low self-esteem for students in the freshman and sophomore years led to low academic self-efficacy 1 year later, which mostly verified the Hypothesis I and Hypothesis II that students’ self-esteem predicted their subsequent academic self-efficacy, yet not vice versa. Moreover, there were gender differences in the prospective effects, which appeared to be stronger and longer among male students compared with their female counterparts.

## Discussion

The findings of the present study facilitated a deeper understanding of the reciprocal relations between self-esteem and academic self-efficacy in the Chinese context. In particular, the four-wave cross-lagged design allowed us to recognize the heterogeneity of the effects across years. Generally, the majority of students enter colleges and universities after taking the National College Entrance Examination (NCEE), and students are usually required to obtain high scores in order to be admitted into their dream schools, which makes the examination of great significance (Ha et al., 2020). Under this circumstance, the exam-oriented patterns of education have been constructed for high school students since they need to make sufficient preparation for the NCEE (Chen and Zou, 2018). In this way, the self-esteem, as well as the academic self-efficacy of freshman students in college, may arise from their performance in NCEE. Meanwhile, considering the fact that most freshman students may still regard academic tasks as their priority in their first year at college by following similar learning modes at high school, their self-esteem and academic self-efficacy should be tightly associated. Nonetheless, the sophomore and junior students are confronted with more demanding tasks not only from the increasing difficulties in class, but also from other aspects such as the establishment of interpersonal networks, the engagement in social activities and internships; while the seniors have to prepare for their future after graduation, e.g., completing graduation thesis, preparing for the postgraduate entrance examination, or seeking jobs in the labor market. Therefore, students in the later years started to detach their self-esteem and academic self-efficacy to avoid possible academic failure, which was evidenced by prior research (Okech and Harrington, 2002).

Another implication of the results pertains to the gender differences in the prospective effect of self-esteem on academic self-efficacy. Our study found that the effect endured longer and stronger among male students, and the possible explanation for this finding is that high esteem among female students brings in not only efforts for academic progress, but also growing interests in making dress codes and wearing makeup (Tran et al., 2020).

The study emphasizes the crucial role of self-esteem on academic self-efficacy for college students in China. Given that the average levels of self-esteem experienced a downward trend over the four academic years, and that the prospective effects of self-esteem on academic self-efficacy weakened over time, we suggest that universities should design relevant educational activities with the purpose of building lasting self-esteem for college students, especially for the freshmen (Afari et al., 2012; Di Giunta et al., 2013; Weisskirch, 2018), which may lead to sustained academic self-efficacy during college life; while students should regard their previous academic achievements in a more positive way in order to gradually strengthen their confidence and self-esteem (Lane et al., 2004; Bhatt and Bahadur, 2018).

In sum, the study evidenced the prospective effect of self-esteem on academic self-efficacy among college students with a four-wave cross-lagged model, which adds to the literature by clarifying the directionality of the relationship between self-esteem and academic self-efficacy in the Chinese context. Meanwhile, the findings also help the higher educational institutions to make proper strategies in order to bolster students’ academic achievement. Future studies can improve the self-report measure of self-esteem and academic self-efficacy by collecting data on students’ behavior and employing big data analytics. The underlying mechanisms of self-esteem and academic self-efficacy can also be explored. In addition, randomized experiments can be designed to evaluate the effects of certain interventions by colleges and universities.

## Limitations

Limitations of this study include the representativeness of the sample. The analysis of our study was based on the students in Beijing, which, as the capital of China, possesses the largest number of universities nationwide. Usually, students enrolled into universities in Beijing are required to score higher compared with those who entered universities of the same tier in other provinces. Thus, the conclusions should be cautiously generalized. In addition, the variables in this study were measured with self-reported instrument, which may result in common method bias.

## Conclusion

First, the average score of academic self-efficacy among Chinese college students declined gradually in the first 3 years before rising slightly in the graduation year. Male students had higher academic self-efficacy than females in the freshman and sophomore years, whereas female students’ academic self-efficacy surpassed their male counterparts in the junior and senior years.

Second, students’ academic self-efficacy was significantly positively associated with their self-esteem.

Third, the self-esteem of college students consistently predicted their subsequent academic self-efficacy, though the effects weakened over time. There were gender differences in the prospective effects from self-esteem to academic self-efficacy, which endured longer and appeared to be stronger among male students compared with their female counterparts.

## Data Availability Statement

The data analyzed in this study is subject to the following licenses/restrictions: the data ownership belongs to the National Survey Research Center, Renmin University of China, Haidian, Beijing, China. The dataset has been used by many publicly published papers with its reliability verified. Researchers interested in this field can also contact the authors should there be any difficulties in data access for academic purposes. The authors will actively meet relevant needs to ensure the replicability and transparency of this study. Requests to access these datasets should be directed to XL, xinqiaoliu@pku.edu.cn.

## Ethics Statement

Ethical review and approval were not required for the study on human participants in accordance with the local legislation and institutional requirements. The patients/participants provided their written informed consent to participate in this study.

## Author Contributions

YL and XL contributed to conception and design of the study. XL and WG undertook the statistical analysis. WG and YL managed the literature searches and analyses. WG, YL, and XL wrote the first draft of the manuscript. All authors contributed to manuscript revision, read, and approved the submitted version.

## Conflict of Interest

The authors declare that the research was conducted in the absence of any commercial or financial relationships that could be construed as a potential conflict of interest.

## Publisher’s Note

All claims expressed in this article are solely those of the authors and do not necessarily represent those of their affiliated organizations, or those of the publisher, the editors and the reviewers. Any product that may be evaluated in this article, or claim that may be made by its manufacturer, is not guaranteed or endorsed by the publisher.

## References

[B1] Afari E. Ward G. Khine M. S. (2012). Global self-esteem and self-efficacy correlates: relation of academic achievement and self-esteem among emirati students. *Int. Educ. Stud.* 5 49–57.

[B2] Ahmadi S. (2020). Academic self-esteem, academic self-efficacy and academic achievement: a path analysis. *J. Foren. Psy.* 5 10–35248.

[B3] Ahmed U. Umrani W. A. Qureshi M. A. Samad A. (2018). Examining the links between teachers support, academic efficacy, academic resilience, and student engagement in bahrain. *Int. J. Adv. Appl. Sci.* 5 39–46. 10.21833/ijaas.2018.09.008

[B4] Ansary N. S. Luthar S. S. (2009). Distress and academic achievement among adolescents of affluence: a study of externalizing and internalizing problem behaviors and school performance. *Dev. Psychopathol.* 21 319–341. 10.1017/S0954579409000182 19144236 PMC4358764

[B5] Arshad M. Zaidi S. M. I. H. Mahmood K. (2015). Self-esteem & academic performance among university students. *J. Educ. Pract.* 6 156–162.

[B6] Bandura A. Freeman W. H. Lightsey R. (1999). Self-efficacy: the exercise of control. *J. Cogn. Psychother*. 13, 158–166. 10.1891/0889-8391.13.2.158 11261958

[B7] Baumeister R. F. Campbell J. D. Krueger J. I Vohs K. D. (2003). Does high self-esteem cause better performance, interpersonal success, happiness, or healthier lifestyles? *Psychol. Sci. Public Int.* 4 1–44. 10.1111/1529-1006.01431 26151640

[B8] Bhatt S. Bahadur A. (2018). Importance of self esteem & self efficacy for college students. *Indian J. Commun. Psychol.* 14 409–419.

[B9] Chen R. Zou M. (2018). “Discussion on the relationship between exam-oriented education and quality-oriented education,” in *Proceeding of the 2018 2nd International Conference on Education Innovation and Social Science (ICEISS 2018).* (Atlantis Press), 350–353.

[B10] Cvencek D. Fryberg S. A. Covarrubias R. Meltzoff A. N. (2018). Self-concepts, self-esteem, and academic achievement of minority and majority north American elementary school children. *Child Dev.* 89 1099–1109. 10.1111/cdev.12802 28386954

[B11] Di Giunta L. Alessandri G. Gerbino M. Kanacri P. L. Zuffiano A. Caprara G. V. (2013). The determinants of scholastic achievement: the contribution of personality traits, self-esteem, and academic self-efficacy. *Learn. Indiv. Diff.* 27 102–108. 10.1348/2044-8279.002004 21199485

[B12] Entwisle D. Kabbani N. Alexander K. (2001). The dropout process in life course perspective: early risk factors at home and school. *Teachers College Record* 103 760–822. 10.1177/016146810110300502

[B13] Gao W. Luo Y. Cao X. Liu X. (2022). Gender differences in the relationship between self-esteem and depression among college students: a cross-lagged study from China. *J. Res. Personali.* 2022:104202. 10.1016/j.jrp.2022.104202

[B14] Gao W. Ping S. Liu X. (2020). Gender differences in depression, anxiety, and stress among college students: a longitudinal study from China. *J. Affect. Dis.* 263 292–300. 10.1016/j.jad.2019.11.121 31818792

[B15] Ha W. Kang L. Song Y. (2020). College matching mechanisms and matching stability: evidence from a natural experiment in China. *J. Econ. Behav. Organiz.* 175 206–226. 10.1016/j.jebo.2020.05.002

[B16] Harris S. L. (2009). *The Relationship Between Self-esteem and Academic Success Among African American Students in the Minority Engineering Program at a Research Extensive University in the Southern Portion of the United States*. LSU Doctoral dissertations, 1726. Available online at: https://digitalcommons.lsu.edu/gradschool_dissertations/1726

[B17] Hawi N. S. Samaha M. (2017). The relations among social media addiction, self-esteem, and life satisfaction in university students. *Soc. Sci. Comput. Rev.* 35 576–586. 10.1177/0894439316660340

[B18] Huang C. (2012). Discriminant and incremental validity of self-concept and academic self-efficacy: a meta-analysis. *Educ. Psychol.* 32 777–805. 10.1080/01443410.2012.732386

[B19] Jiang Y. Song J. Lee M. Bong M. (2014). Self-efficacy and achievement goals as motivational links between perceived contexts and achievement. *Educ. Psychol.* 34 92–117. 10.3389/fpsyg.2021.746608 34744920 PMC8564472

[B20] Joshanloo M. Afshari S. (2011). Big five personality traits and self-esteem as predictors of life satisfaction in iranian muslim university students. *J. Happ. Stud.* 12 105–113. 10.1007/s10902-009-9177-y

[B21] Kirkpatrick R. Zang Y. (2011). The negative influences of exam-oriented education on chinese high school students: backwash from classroom to child. *Lang. Test. Asia* 1 1–10.

[B22] Klassen R. M. Usher E. L. (2010). “Self-efficacy in educational settings: Recent research and emerging directions,” in *The Decade Ahead: Theoretical Perspectives on Motivation and Achievement*, Vol. 16(Pt A), eds Urdan T. C. Karabenick S. A. (Bingley: Emerald Group Publishing Limited), 1–33. 10.1108/S0749-7423(2010)000016A004

[B23] Kong F. Zhao J. You X. (2012). Emotional intelligence and life satisfaction in chinese university students: the mediating role of self-esteem and social support. *Personali. Indiv. Diff.* 53 1039–1043. 10.1016/j.paid.2012.07.032

[B24] Kuh G. D. Kinzie J. L. Buckley J. A. Bridges B. K. Hayek J. C. (2006). *What Matters to Student Success: A Review of the Literature*, Vol. 8. Washington, DC: National Postsecondary Education Cooperative.

[B25] Lane J. Lane A. M. Kyprianou A. (2004). Self-efficacy, self-esteem and their impact on academic performance. *Soc. Behav. Personali. Int. J.* 32 247–256. 10.2224/sbp.2004.32.3.247

[B26] Li J. Han X. Wang W. Sun G. Cheng Z. (2018). How social support influences university students’ academic achievement and emotional exhaustion: the mediating role of self-esteem. *Learn. Indiv. Diff.* 61 120–126. 10.1016/j.lindif.2017.11.016

[B27] Lin S. H. Lu W. C. Chen M. Y. Chen L. H. (2014). Association between proactive personality and academic self–efficacy. *Curr. Psychol.* 33 600–609. 10.1007/s12144-014-9231-8

[B28] Liu X. Gao X. Ping S. (2019a). Post-1990s college students academic sustainability: the role of negative emotions, achievement goals, and self-efficacy on academic performance. *Sustainability* 11:775. 10.3390/su11030775

[B29] Liu X. Ping S. Gao W. (2019b). Changes in undergraduate students’ psychological well-being as they experience university life. *Int. J. Environ. Res. Public Health* 16:2864. 10.3390/ijerph16162864 31405114 PMC6719208

[B30] Mao Y. Yang R. Bonaiuto M. Ma J. Harmat L. (2020). Can flow alleviate anxiety? The roles of academic self-efficacy and self-esteem in building psychological sustainability and resilience. *Sustainability* 12:2987. 10.3390/su12072987

[B31] Midgley C. Maehr M. L. Hruda L. Z. Anderman E. Anderman L. Freeman K. E. (2000). *Manual for the Patterns Of Adaptive Learning Scales.* Ann Arbor: University of Michigan.

[B32] Okech A. P. Harrington R. (2002). The relationships among black consciousness, self-esteem, and academic self-efficacy in african american men. *J. Psychol.* 136 214–224. 10.1080/00223980209604151 12081095

[B33] Pajares F. Schunk D. H. (2001). Self-beliefs and school success: Self-efficacy, self-concept, and school achievement. *Perception* 11 239–266.

[B34] Parker S. K. Bindl U. K. Strauss K. (2010). Making things happen: a model of proactive motivation. *J. Manage.* 36 827–856. 10.1177/0149206310363732

[B35] Robert R. Lenore J. (1968). *Pygmalion in the Classroom: Teacher Expectation and Pupils’ Intellectual Development*. New York, NY: Rinehart and Winston, Inc.

[B36] Rosenberg M. (1965). Rosenberg self-esteem scale (RSE). Acceptance and commitment therapy. *Measures package* 61:18.

[B37] Saadat M. Ghasemzadeh A. Soleimani M. (2012). Self-esteem in Iranian university students and its relationship with academic achievement. *Procedia Soc. Behav. Sci.* 31 10–14. 10.1016/j.sbspro.2011.12.007

[B38] Shea C. M. Howell J. M. (2000). Efficacy-performance spirals: an empirical test. *J. Manage.* 26 791–812. 10.1177/014920630002600409

[B39] Suprayogi M. N. Ratriana L. Wulandari A. P. J. (2019). The interplay of academic efficacy and goal orientation toward academic achievement. *J. Phys. Conf. Ser.* 1175:012132. 10.1088/1742-6596/1175/1/012132

[B40] Talsma K. Schüz B. Schwarzer R. Norris K. (2018). I believe, therefore I achieve (and vice versa): a meta-analytic cross-lagged panel analysis of self-efficacy and academic performance. *Learn. Indiv. Diff.* 61 136–150. 10.1016/j.lindif.2017.11.015

[B41] Tran A. Rosales R. Copes L. (2020). Paint a better mood? Effects of makeup use on youtube beauty influencers’ self-esteem. *Sage Open* 10:2158244020933591.

[B42] Weisskirch R. S. (2018). Grit, self-esteem, learning strategies and attitudes and estimated and achieved course grades among college students. *Curr. Psychol.* 37 21–27. 10.1007/s12144-016-9485-4

